# Interrelations between blood-brain barrier permeability and matrix metalloproteinases are differently affected by tissue plasminogen activator and hyperoxia in a rat model of embolic stroke

**DOI:** 10.1186/2045-9912-2-2

**Published:** 2012-01-24

**Authors:** Dominik Michalski, Carsten Hobohm, Christopher Weise, Johann Pelz, Marita Heindl, Manja Kamprad, Johannes Kacza, Wolfgang Härtig

**Affiliations:** 1 Department of Neurology, University of Leipzig, Liebigstr. 20, 04103 Leipzig, Germany; 2 Paul Flechsig Institute for Brain Research, University of Leipzig, Jahnallee 59, 04109 Leipzig, Germany; 3 Institute of Clinical Immunology and Transfusion Medicine, University of Leipzig, Johannisallee 30, 04103 Leipzig, Germany; 4 Department of Anatomy, Histology and Embryology, Faculty of Veterinary Medicine, University of Leipzig, An den Tierkliniken 43, 04103 Leipzig, Germany

**Keywords:** Experimental stroke, blood-brain barrier, FITC-albumin, MMP, TIMP, tissue plasminogen activator, NBO, HBO

## Abstract

**Background:**

In ischemic stroke, blood-brain barrier (BBB) regulations, typically involving matrix metalloproteinases (MMPs) and inhibitors (TIMPs) as mediators, became interesting since tissue plasminogen activator (tPA)-related BBB breakdown with risk of secondary hemorrhage was considered to involve these mediators too. Despite high clinical relevance, detailed interactions are purely understood. After a pilot study addressing hyperoxia as potential neuroprotective co-treatment to tPA, we analyzed interrelations between BBB permeability (BBB-P), MMPs and TIMPs.

**Findings:**

Rats underwent embolic middle cerebral artery occlusion (eMCAO) and treatment with normobaric (NBO) or hyperbaric oxygen (HBO), tPA, tPA+HBO, or no treatment. BBB-P was assessed by intravenously applied FITC-albumin at 4 or 24 hours. MMP-2/-9 and TIMP-1/-2 serum levels were determined at 5 or 25 hours. Time point-corrected partial correlations were used to explore interrelations of BBB-P in ischemic regions (extra-/intravasal FITC-albumin ratio) and related serum markers. BBB-P correlated positively with MMP-2 and MMP-9 in controls, whereas hyperoxia led to an inverse association, most pronounced for HBO/MMP-9 (r = -0.606; *P *< 0.05). As expected, positive coefficients were observed after treatment with tPA. Co-treatment with HBO attenuated and in part reversed this effect, but to a lower degree than HBO alone. Amongst MMPs and TIMPs, significant associations shifted from MMP-9 to -2 when comparing treatment with HBO/tPA and tPA+HBO. TIMPs were significantly interrelated after tPA, tPA+HBO, and interestingly, HBO alone.

**Conclusions:**

HBO was found to reverse the positively directed interrelation of BBB-P and MMPs after eMCAO, but this effect failed to sustain in the expected amount when HBO and tPA were given simultaneously.

## Findings

Multiple clinical trials have proven the efficacy of tissue plasminogen activator (tPA) in the treatment of acute focal cerebral ischemia [1]. Apart from its beneficial recanalizing actions, tPA has also detrimental effects contributing to blood-brain barrier (BBB) disruption in ischemia-affected brain tissue [2-4] with an increased risk of secondary hemorrhage and poor outcome [5,6]. Enzymes, e.g., matrix metalloproteinases (MMPs) and their inhibitors (TIMPs), are considered as key factors regulating the BBB integrity [7,8]. Thereby, MMPs possess both destructive abilities in earlier phases of ischemia and regenerative properties (e.g., remodeling) in later phases [9], which implicate complex interactions in the time course of ischemic stroke. Interestingly, tPA was found to directly increase MMP levels in experimental and clinical stroke [4,10-12], which is considered as a main tPA-related mechanism of BBB breakdown [3,4,13]. This led to increasing efforts to develop neuroprotective strategies with the aim to selectively attenuate tPA side effects [14]. In this regard, treatment with hyperoxia - the application of 100% oxygen under normal (normobaric oxygen; NBO) or elevated ambient pressure (hyperbaric oxygen; HBO) [15] - was discussed as potential co-treatment [16]. This rationale based on the reported positive effects of hyperoxia in experimental stroke, e.g., reduced degradation of BBB components, inhibition of MMP-9 upregulation [17], and decreased secondary hemorrhage when tPA was given following hyperoxia [18,19]. Our group has recently demonstrated that hyperoxia, tPA and especially their simultaneous application differentially affects BBB permeability and alters plasma concentrations of MMPs and TIMPs [20].

Considering the potential clinical use of hyperoxia in acute ischemic stroke - especially in combination with tPA - we aimed to further clarify mechanisms of ischemia-related BBB alterations including the role of involved MMPs and TIMPs. Here, we tested the hypothesis that tPA and hyperoxia (i.e. NBO or HBO) influence the interrelations between BBB permeability, MMP-2/-9 and TIMP-1/-2 in the early phase of experimental focal cerebral ischemia.

For this purpose, we retrospectively analyzed data from our previous work [20]. The underlying study and the associated experimental animals procedures were approved by local authorities and conducted according to the 86/609/EEC. In detail, male Wistar rats underwent focal cerebral ischemia by right-sided embolic middle cerebral artery occlusion (eMCAO) as described previously [20,21]. Two hours after eMCAO, the animals were treated with 60 minutes of NBO or HBO (2.4 absolute atmospheres) in a special oxygen chamber (Sayers/Hebold, Cuxhaven, Germany), tPA (Actilyse, Boehringer, Ingelheim, Germany; 9 mg/kg body weight intravenously over 30 minutes), combined tPA and HBO (tPA+HBO), or received no treatment (control). Four or 24 hours after ischemia onset *via *eMCAO, 20 mg of FITC-albumin (Sigma, Taufkirchen, Germany; in 1 mL physiological saline solution [NaCl]) was administered intravenously. After an additional circulation period of usually 1 hour, the animals were deeply anesthetized and blood samples for MMP-2, -9, TIMP-1 and -2 serum concentrations were obtained transcardially, followed by perfusion with NaCl and 4% paraformaldehyde in phosphate-buffered saline. Tissue preparation and quantification of BBB integrity in ischemia-affected brain regions using FITC-albumin as permeability marker were described previously [20,22]. The resulting extra-/intravasal ratio was utilized for calculations in the present study. Overall, data from 78 animals were used for statistical analyses of interrelations between the extra-/intravasal FITC-albumin ratio as surrogate for ischemia-related BBB permeability, and serum concentrations of MMPs and TIMPs *via *time point-corrected (survival period) partial correlations. Calculations were made with SPSS 18.0 (SPSS Inc., an IBM Company, Chicago, IL); a *P *< 0.05 was considered statistically significant.

**Figure 1:**
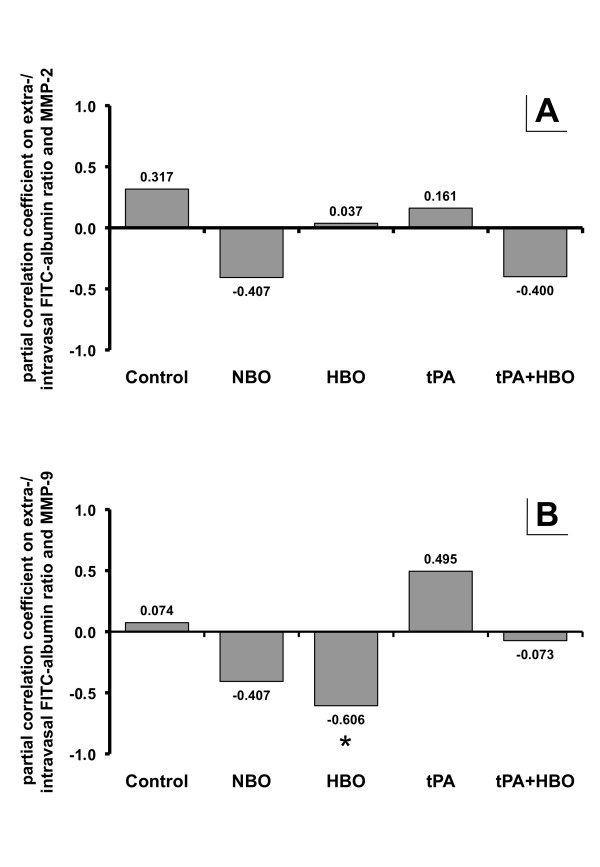
**Time point-corrected partial correlation coefficients between the extra-/intravasal FITC-albumin ratio as surrogate for blood-brain barrier permeability in ischemia-affected brain regions and matrix metalloproteinase (MMP)-2 (A) as well as MMP-9 (B) serum levels depending on treatment**. Underlying degrees of freedom *(df)*: In both (A) and (B) for controls n = 13, for NBO n = 13, for HBO n = 12, for tPA n = 11, and for tPA+HBO n = 13. Coefficient significance level: *: *P *< 0.05.

We first focused on interrelations between the ischemia-induced changes in BBB permeability, MMP-2 and -9 in controls (Figure 1) to explore the natural pathophysiology in the applied model of experimental stroke. Non-significant but positive partial correlation coefficients were observed for both MMPs investigated, particularly MMP-2 (r = 0.317), indicating an unidirectional association between BBB permeability and plasma MMP concentrations. Figure 1 further displays the results after treatment with hyperoxia (NBO or HBO), tPA and its combination with HBO. While we observed in NBO-treated animals negative correlation coefficients for both MMP-2 and -9, HBO resulted in a significant negative correlation (r = 0.606; *P *= 0.022) for MMP-9, representing an inverse association between BBB permeability and MMP levels. As expected, treatment with tPA led to unidirectional associations of BBB permeability and MMP levels as indicated by positive correlation coefficients, again pronouncing MMP-9. Interestingly, treatment with tPA+HBO resulted in inverse associations between BBB permeability and both MMPs when compared to tPA alone, but this effect was much lower as in groups treated with NBO or HBO alone. Second, we focused on interrelations between MMPs and TIMPs in all animal groups exploring the natural relationship of these enzymes in untreated focal cerebral ischemia (Table 1), and after different treatments (Table 2 and 3). In controls, non-significant correlation coefficients were found, although values ranged from -0.314 (MMP-9 and TIMP-2) to 0.345 (MMP-2 and TIMP-2). A comparable situation was noted in NBO-treated animals (Table 2, upper right), but interestingly, HBO led to interrelations with significant coefficients (Table 2, lower left). Thereby, almost identical positive correlation coefficients of about 0.7 were obtained between TIMP-1 and MMP-9, TIMP-2 and MMP-9 as well as TIMP-1 and -2, clearly indicating treatment-specific alterations when compared to controls (Table 1). For tPA (Table 3, upper right), significant coefficients of about 0.7 were detected between TIMP-2 and MMP-9 as well as TIMP-1 and -2. In contrast, administration of tPA+HBO (Table 2, lower left) did not affect the MMP-9 route significantly, but provided significant and positively directed correlation coefficients (about 0.6 and 0.8) between MMP-2 and TIMP-1 as well as TIMP-2. Notably, TIMP-1 and -2 were also significantly positively correlated after combined treatment with tPA and HBO, which has already been noted for tPA and HBO alone with nearly the same coefficients (about 0.76).

**Table 1:** Time point-corrected partial correlation coefficients between BBB-related serum markers in control animals.

	MMP-2	MMP-9	TIMP-1	TIMP-2
**MMP-2**	-	0.199 *P *= 0.478	0.083 *P *= 0.768	0.345 *P *= 0.207
**MMP-9**		-	-0.055 *P *= 0.844	-0.314 *P *= 0.254
**TIMP-1**			-	0.302 *P *= 0.274
**TIMP-2**				-

Underlying degrees of freedom *(df)*: n = 13.

**Table 2:** Time point-corrected partial correlation coefficients between BBB-related serum markers in animals treated with NBO (upper right) or HBO (lower left).

	MMP-2	MMP-9	TIMP-1	TIMP-2
**MMP-2**	-	0.240 *P *= 0.389	0.217 *P *= 0.437	0.071 *P *= 0.801
**MMP-9**	0.130 *P *= 0.657	-	-0.161 *P *= 0.567	0.341 *P *= 0.214
**TIMP-1**	0.012 *P *= 0.967	**0.710** ***P *= 0.004**	-	0.024 *P *= 0.932
**TIMP-2**	0.085 *P *= 0.773	**0.626** ***P *= 0.017**	**0.764** ***P *= 0.001**	-

Underlying degrees of freedom *(df)*: for NBO n = 13, for HBO n = 12.

**Table 3:** Time point-corrected partial correlation coefficients between BBB-related serum markers in animals treated with tPA alone (upper right) or tPA+HBO (lower left).

	MMP-2	MMP-9	TIMP-1	TIMP-2
**MMP-2**	-	0.277 *P *= 0.360	0.325 *P *= 0.279	0.445 *P *= 0.128
**MMP-9**	-0.045 *P *= 0.873	-	0.425 *P *= 0.148	**0.725** ***P *= 0.005**
**TIMP-1**	**0.628** ***P *= 0.012**	0.155 *P *= 0.580	-	**0.757** ***P *= 0.003**
**TIMP-2**	**0.834** ***P *= 0.000**	0.306 *P *= 0.268	**0.760** ***P *= 0.001**	-

Underlying degrees of freedom *(df)*: for tPA n = 11, for tPA+HBO n = 13.

Despite its retrospective design, the present study provided clinically relevant insights in interactions of stroke-related BBB permeability with associated MMPs and TIMPs in consideration of tPA as approved treatment for ischemic stroke [1], and hyperoxia as potential neuroprotective co-treatment [16]. Thereby, HBO was chosen since a previous report has already tested the combination of NBO and tPA [23]. Concerning translational aspects, efforts were made to use an embolic model for focal cerebral ischemia, representing probably the best comparability to the human pathophysiology [24]. Furthermore, BBB-related changes based on the leakage marker FITC-albumin and BBB-associated serum markers were assessed simultaneously, which required specific brain tissue preparations for immunofluorecence labeling [22] and significant correlations between plasma and brain levels of MMPs, as shown by a previous study for MMP-9 [25].

The first main finding of the present study was the ability of hyperoxia to reverse the (in controls positively directed) association of BBB permeability, MMP-2 and -9 when administered 2 hours after ischemia onset. This effect was most pronounced for MMP-9 and treatment with HBO, suggesting a specific influence of HBO on BBB regulation. Based on the presented data the previously reported tendency for BBB stabilization following HBO administration 2 hours after eMCAO [20] might be attributed to an interruption of the MMP-9-related increase of BBB permeability. This view is supported by former studies showing an attenuated MMP-9 upregulation after experimental stroke treated with hyperoxia [17,19]. As already known from earlier reports, treatment with tPA leads to an increase in BBB permeability that is mainly caused by MMP-9 activation [2,10,12], which was confirmed by our data (positive correlation coefficient between BBB permeability and MMP-9). Concerning the potential neuroprotective effect of HBO when simultaneously applied to tPA and its clinical potential, we found that this approach attenuated, and actually reversed the effect of tPA in part, but this effect was less pronounced than after treatment with HBO alone. However, these data suggest that HBO simultaneously applied to tPA starting 2 hours after experimental stroke does not result in the assumed neuroprotective effect *via *a significant prevention of the MMP-9-related increase in BBB permeability. Moreover, this finding should be seen in context with our previous study [20] which noted a tendency towards increased BBB permeability and plasma concentrations of MMPs and TIMPs when addressed separately and, therefore, opens up a critical perspective on this combined approach.

The second part of the present study addressed interrelations of MMPs and TIMPs. Here, we found mainly week and non-significant associations in controls and NBO-treated animals. This situation clearly changed after treatment with HBO, tPA and the combined approach: HBO and tPA led to significant positive associations between MMP-9 and TIMP-2, and in part MMP-9 and TIMP-1. However, treatment with tPA+HBO resulted in a shift towards significant positive associations between MMP-2 and TIMP-1/-2, while MMP-9 provided non-significant coefficients. Interestingly, a significant correlation coefficient between TIMP-1 and -2 was found for all types of treatment with the exception of NBO. Why this association was also present for HBO without concomitant tPA treatment remains open and requires further evaluation. Unfortunately, a clear interpretation of these mediator-related changes is still impeded by the complexity of MMP activation, including the expression of pro-MMPs, the interference with other MMPs (e.g., MMP-3) [4], and non-selective inhibitory processes by TIMPs [8] with reported positive and negative effects depending on the time course of ischemia [3].

## Competing interests

The authors declare that they have no competing interests.

## Authors' contributions

DM, CH, JK and WH designed the underlying study. DM and CW carried out animal experiments; JP and WH performed tissue preparation and serial staining. JP assessed FITC-albumin extravasation and MK measured serum MMPs and TIMPs, both in a blinded manner. DM analyzed the data and wrote the manuscript, WH, CW, MH and CH made critical revisions. All authors have read and approved the submitted manuscript.

## References

[B1] Hacke W Kaste M Bluhmki E Brozman M Dávalos A Guidetti D Larrue V Lees KR Medeghri Z Machnig T Schneider D von Kummer R Wahlgren N Toni D ECASS Investigators Thrombolysis with alteplase 3 to 4.5 hours after acute ischemic stroke N Engl J Med 2008 359 1317 1329 10.1056/NEJMoa0804656 18815396

[B2] Kelly MA Shuaib A Todd KG Matrix metalloproteinase activation and blood-brain barrier breakdown following thrombolysis Exp Neurol 2006 200 38 49 10.1016/j.expneurol.2006.01.032 16624294

[B3] Adibhatla RM Hatcher JF Tissue plasminogen activator (tPA) and matrix metalloproteinases in the pathogenesis of stroke: therapeutic strategies CNS Neurol Disord Drug Targets 2008 7 243 253 10.2174/187152708784936608 18673209 PMC2562687

[B4] Jin R Yang G Li G Molecular insights and therapeutic targets for blood-brain barrier disruption in ischemic stroke: critical role of matrix metalloproteinases and tissue-type plasminogen activator Neurobiol Dis 2010 38 376 385 10.1016/j.nbd.2010.03.008 20302940 PMC2862862

[B5] Ergul A Elgebaly MM Middlemore ML Li W Elewa H Switzer JA Hall C Kozak A Fagan SC Increased hemorrhagic transformation and altered infarct size and localization after experimental stroke in a rat model type 2 diabetes BMC Neurol 2007 7 33 10.1186/1471-2377-7-33 17937795 PMC2098774

[B6] Gumbinger C Gruschka P Böttinger M Heerlein K Barrows R Hacke W Ringleb P Improved prediction of poor outcome after thrombolysis using conservative definitions of symptomatic hemorrhage Stroke 2012 43 240 242 10.1161/STROKEAHA.111.623033 21998049

[B7] Cunningham LA Wetzel M Rosenberg GA Multiple roles for MMPs and TIMPs in cerebral ischemia Glia 2005 50 329 339 10.1002/glia.20169 15846802

[B8] Candelario-Jalil E Yang Y Rosenberg GA Diverse roles of matrix metalloproteinases and tissue inhibitors of metalloproteinases in neuroinflammation and cerebral ischemia Neuroscience 2009 158 983 994 10.1016/j.neuroscience.2008.06.025 18621108 PMC3584171

[B9] Rosell A Lo EH Multiphasic roles for matrix metalloproteinases after stroke Curr Opin Pharmacol 2008 8 82 89 10.1016/j.coph.2007.12.001 18226583

[B10] Burggraf D Martens HK Dichgans M Hamann GF rt-PA causes a dose-dependent increase in the extravasation of cellular and non-cellular blood elements after focal cerebral ischemia Brain Res 2007 1164 55 62 17644075 10.1016/j.brainres.2007.05.066

[B11] Aoki T Sumii T Mori T Wang X Lo EH Blood-brain barrier disruption and matrix metalloproteinase-9 expression during reperfusion injury: mechanical versus embolic focal ischemia in spontaneously hypertensive rats Stroke 2002 33 2711 2717 10.1161/01.STR.0000033932.34467.97 12411666

[B12] Tsuji K Aoki T Tejima E Arai K Lee SR Atochin DN Huang PL Wang X Montaner J Lo EH Tissue plasminogen activator promotes matrix metalloproteinase-9 upregulation after focal cerebral ischemia Stroke 2005 36 1954 1959 10.1161/01.STR.0000177517.01203.eb 16051896

[B13] Morancho A Rosell A García-Bonilla L Montaner J Metalloproteinase and stroke infarct size: role for anti-inflammatory treatment? Ann N Y Acad Sci 2010 1207 123 133 10.1111/j.1749-6632.2010.05734.x 20955435

[B14] Endres M Engelhardt B Koistinaho J Lindvall O Meairs S Mohr JP Planas A Rothwell N Schwaninger M Schwab ME Vivien D Wieloch T Dirnagl U Improving outcome after stroke: overcoming the translational roadblock Cerebrovasc Dis 2008 25 268 278 10.1159/000118039 18292653

[B15] Jain KK Textbook of Hyperbaric Medicine 2009 5 Göttingen: Hogrefe & Huber

[B16] Singhal AB A review of oxygen therapy in ischemic stroke Neurol Res 2007 29 173 183 10.1179/016164107X181815 17439702

[B17] Veltkamp R Bieber K Wagner S Beynon C Siebing DA Veltkamp C Schwaninger M Marti HH Hyperbaric oxygen reduces basal lamina degradation after transient focal cerebral ischemia in rats Brain Res 2006 1076 231 237 10.1016/j.brainres.2006.01.013 16480689

[B18] Sun L Zhou W Mueller C Sommer C Heiland S Bauer AT Marti HH Veltkamp R Oxygen therapy reduces secondary hemorrhage after thrombolysis in thromboembolic cerebral ischemia J Cereb Blood Flow Metab 2010 30 1651 1660 10.1038/jcbfm.2010.50 20424638 PMC2949252

[B19] Liu W Hendren J Qin XJ Liu KJ Normobaric hyperoxia reduces the neurovascular complications associated with delayed tissue plasminogen activator treatment in a rat model of focal cerebral ischemia Stroke 2009 40 2526 2531 10.1161/STROKEAHA.108.545483 19478225 PMC2714253

[B20] Michalski D Pelz J Weise C Kacza J Boltze J Grosche J Kamprad M Schneider D Hobohm C Härtig W Early outcome and blood-brain barrier integrity after co-administered thrombolysis and hyperbaric oxygenation in experimental stroke Exp Transl Stroke Med 2011 3 5 10.1186/2040-7378-3-5 21679435 PMC3144445

[B21] Zhang RL Chopp M Zhang ZG Jiang Q Ewing JR A rat model of focal embolic cerebral ischemia Brain Res 1997 766 83 92 10.1016/S0006-8993(97)00580-5 9359590

[B22] Michalski D Grosche J Pelz J Schneider D Weise C Bauer U Kacza J Gärtner U Hobohm C Härtig W A novel quantification of blood-brain barrier damage and histochemical typing after embolic stroke in rats Brain Res 2010 1359 186 200 20732314 10.1016/j.brainres.2010.08.045

[B23] Fujiwara N Murata Y Arai K Egi Y Lu J Wu O Singhal AB Lo EH Combination therapy with normobaric oxygen (NBO) plus thrombolysis in experimental ischemic stroke BMC Neurosci 2009 10 79 10.1186/1471-2202-10-79 19604385 PMC2714858

[B24] Young AR Ali C Duretête A Vivien D Neuroprotection and stroke: time for a compromise J Neurochem 2007 103 1302 1309 10.1111/j.1471-4159.2007.04866.x 17727635

[B25] Park KP Rosell A Foerch C Xing C Kim WJ Lee S Opdenakker G Furie KL Lo EH Plasma and brain matrix metalloproteinase-9 after acute focal cerebral ischemia in rats Stroke 2009 40 2836 2842 10.1161/STROKEAHA.109.554824 19556529 PMC3712850

